# Supporting Newly Identified or Diagnosed Autistic Adults: An Initial Evaluation of an Autistic-Led Programme

**DOI:** 10.1007/s10803-020-04486-4

**Published:** 2020-04-07

**Authors:** Laura Crane, Caroline Hearst, Maria Ashworth, Jade Davies, Elisabeth L. Hill

**Affiliations:** 1 Present Address: Centre for Research in Autism and Education (CRAE), UCL Institute of Education, University College London, 55-59 Gordon Square, London, WC1H 0NU UK; 2 AutAngel CIC and Autism Matters, Reading, UK; 3 Department of Psychology, Goldsmiths, University of London, London, UK

**Keywords:** Autism, Adults, Diagnosis, Post-diagnostic support, Autistic-led, Peer support

## Abstract

Sixteen adults (diagnosed or self-identified as autistic) participated in one of two iterations of a ten-week autistic-led programme, aimed at helping autistic adults learn more about autism within a peer group context. Motivations for taking part in the programme included a desire for: (1) exploration of autism; (2) empowerment; and (3) the development of practical strategies and coping mechanisms. Interviews were conducted upon completion of the programme and again 6 months later. Using thematic analysis, three themes were identified: (1) appreciation of the autistic-led nature of the programme; (2) unity in diversity; and (3) developing a positive, practical outlook on autism. These promising initial results highlight the value of autistic-led peer support for those recently diagnosed/identified as autistic.

## Introduction

Our conceptions of autism have changed over time. Whilst early accounts suggested that autism was a childhood condition, largely affecting those with associated challenges in language and intellectual functioning (e.g., Kanner 1943), the spectrum of autism was subsequently widened to include those who met the core criteria for an autism diagnosis, but who did not have co-occurring intellectual disability and/or early language delays (who were, until recently, considered to have ‘high functioning’ autism^1^ or Asperger syndrome) (Hansen et al. 2015; Fletcher-Watson and Happé 2019). Consequently, many adults have been identified as autistic^2^ later in life, having slipped through the diagnostic net in childhood (Happé et al. 2016). This is particularly true for people who may not conform to traditional, stereotypical descriptions of autism (e.g., women and girls), and may be particularly vulnerable to missed (or mis-) diagnosis (Bargiela et al. 2016; Gould and Ashton-Smith 2011; Leedham et al. 2019).

The identification of autism (either formally or informally) can have a huge impact on the life of a person and those close to them; particularly if identification first occurs in adulthood. After years of not ‘fitting in’, autistic adults often report relief (and even elation) in finally having an explanation for their feelings of difference (Hearst 2019; Williams 2019). Moreover, diagnosis in adulthood can lead to better self-awareness, and an appreciation of personal needs (Stagg and Belcher 2019). For some, this identification may come as a complete surprise: commonly stemming from an assessment for another condition (e.g., a mental health diagnosis) or following their children receiving an autism diagnosis (Crane et al. 2018). For others, the formal confirmation of an autism diagnosis can be important in validating suspicions that they are on the autistic spectrum (Hearst 2019). In either case, the diagnosis can be an important gateway to support and services (Crane et al. 2018). Yet there are many barriers to accessing a formal autism diagnosis in adulthood, including lengthy delays (Jones et al. 2014) and a fear of not being believed by professionals (Lewis 2017). These barriers may be particularly pronounced for certain groups, such as women and girls (Bargiela et al. 2016), those from minority ethnic communities (e.g., Zuckerman et al. 2014) and those without intellectual disabilities and/or early language delays (Crane et al. 2016). Indeed, clinical professionals involved in autism diagnosis (e.g., general practitioners, psychologists, psychiatrists) have noted the challenges in identifying and diagnosing autism in members of these groups (e.g., Crane et al. 2019a, b; Rogers et al. 2016; Unigwe et al. 2017).

Following identification or diagnosis of autism, the question often asked by autistic adults is ‘where to from here?’ (Hearst 2019, p. 5). The diagnostic process can be extremely challenging; raising personal and emotional experiences from the past, but not providing the time or space to enable these to be worked through (Crane et al. 2018). Then, following the diagnosis, autistic adults tend to be dissatisfied with the help and support offered, with many receiving no support at all (Crane et al. 2018; Jones et al. 2014). A lack of professional post-diagnostic support may be compounded by little or no family support, for example, due to misunderstandings between the autistic person and their family, or because family members have refused to accept the autistic person’s diagnosis (Crane et al. 2018). Yet post-diagnostic support is crucial, particularly given the range of negative outcomes that autistic adults may face. These include challenges with employment (Shattuck et al. 2012) and social participation (Orsmond et al. 2013); poor mental health (Moss et al. 2015) and quality of life (Ayres et al. 2018); and high rates of premature mortality (Hirvikoski et al. 2016). Whilst various post-diagnostic support programmes have been developed for parents (e.g., EarlyBird; see Dawson-Squibb et al. 2019) and young autistic people (e.g., PEGASUS; see Gordon et al. 2015), there is little equivalent support available for autistic adults.

Post-diagnostic support for autistic adults (as with most information, services and support for this group) tends to be provided by non-autistic professionals. Yet autistic self-advocates, inspired by the disability-rights movement (Shapiro 1994), call for “nothing about us without us”: recognising the need for autistic people themselves to have a central voice in the services and support available to them. Post-diagnostic peer support for autistic adults may be particularly helpful in this regard. There is a growing body of evidence advocating the use of peer support for children on the autistic spectrum (e.g., Gordon et al. 2015). Further, it has been suggested that the social relationship challenges that autistic adults face may best be mitigated by developing relationships with other autistic people (NICE 2012). Advantages of peer support over professional support include greater empathy (given the shared lived experience of autism), gaining hope from seeing peers with the same diagnosis successfully navigating a (largely) neurotypical world, and a greater understanding of autism and how it may manifest in others. Whilst peer support may not replace the need for professional support, it may serve as a useful process for newly diagnosed/identified autistic adults, particularly if it is autistic-led.

In this paper, we report on an initial evaluation of an autistic-led post-identification programme for autistic adults recently identified or diagnosed as autistic. The ten-week programme (*Exploring Being Autistic*) was developed by an autistic consultant and trainer. It aimed to enable people diagnosed or self-identified as autistic to: learn about autism and discover if/how it affects them personally; process emotional response to identification/diagnosis; consider the pros and cons of disclosing that they are autistic; develop strategies to capitalise on the strengths and mitigate the challenges associated with autism; and socialise with peers. In this paper, we report on how the developer of *Exploring Being Autistic* (CH) worked with a team of researchers (led by LC) to conduct a preliminary, qualitative evaluation of the programme. The goals of the evaluation were to identify any benefits of the programme for participants, as well as ways to make the programme more acceptable to participants in future.

## Method

### Design

Two iterations of the *Exploring Being Autistic* programme were evaluated as part of this project: one from May 2016 to July 2016 (with nine participants) and one from September 2016 to November 2016 (with seven participants). Prior to taking part, participants completed a brief questionnaire (gathering qualitative data on their motivations for, and expectations of, taking part in the programme). Participants were then interviewed immediately after the programme, and again 6 months later, generating qualitative data about their experiences. Ethical approval was obtained from the Department of Psychology at Goldsmiths, University of London.

### Participants

Advertisements for *Exploring Being Autistic* were circulated via the website of the organisation running the programme (an autistic-led community interest group for autistic adults). It was also advertised on a local community website and via word of mouth. Interested participants were invited to an informal, one-to-one meeting with the group facilitator, which enabled the facilitator to: get to know the participants; ensure an effective group dynamic; enhance the comfort of participants (so that they knew at least one person prior to attending the group); and to identify any needs that participants had.

In total, 16 autistic adults took part in the programme, across the two iterations. All 16 took part in interviews immediately after the programme, and 11 took part in 6-month follow-up interviews (see Table 1 for details). Participation, in both the programme and the research, was voluntary. For the research, participants were provided with information sheets and offered discussions with the researcher and/or facilitator from which to give informed consent to take part in the interviews. Participation in the programme was not dependent upon participation in the research (a fact emphasised to participants), yet rates of participation were high. All participants took part in the first round of interviews immediately after the programme. Fewer participants from iteration two decided to take part in the follow-up interviews. On the advice of the programme facilitator, the researcher made effort to build up trust with the participants by spending time with them and being willing to explain and answer questions about her motivation and rationale for carrying out the research.

**Table 1:** Participant information

	Pseudonym	Gender	Age	Attended previous support group	Rating (max = 10) for previous support group	Participated in 6 month follow-up interview
Iteration 1	Andrew	Male	71	No	N/A	Yes
	Brooke	Female	53	No	N/A	Yes
	Callum	Male	31	Yes	8	Yes
	Danielle	Female	43	No	N/A	Yes
	Emily	Female	43	No	N/A	Yes
	Fiona	Female	66	Yes	3	Yes
	Grace	Female	47	No	N/A	Yes
	Harry	Male	25	No	N/A	Yes
	Isabelle	Female	33	Yes	6	No
Iteration 2^a^	Jane	Female	45	Yes	7	No
	Kayla	Female	53	Yes	8	Yes
	Lucy	Female	59	No	N/A	Yes
	Matthew	Male	18	No	N/A	No
	Nigel	Male	52	Yes	9	No
	Olivia	Female	32	Yes	7	Yes
	Paula	Female	40	No	N/A	No

The average age of participants was 44.24 years (SD = 14.51), ranging from 18 to 71 years. Nine participants had a formal autism diagnosis and seven self-identified as autistic. Seven participants had attended support groups in the past, and overall experiences of these were fairly positive: on a scale of one to ten (from ‘not at all useful’ to ‘extremely useful’), the average score was 6.57 (SD = 1.90)

^a^Note: originally, there were nine adults in the second iteration of the programme, but one withdrew from the programme due to personal reasons, and another withdrew due to severe anxiety unrelated to the programme

### Materials

#### Pre-programme Questionnaire

Pre-programme questionnaires were used to collect demographic information from participants (e.g., age, gender identity). These were also used to determine whether participants had attended support groups/programmes in the past and, if so, to rate the usefulness of this. Participants were then asked why they wanted to take part in the programme and what they hoped to gain from taking part (in open-ended text boxes). Finally, they were asked whether they had received enough information prior to taking part in the programme and, if not, to state what they would have liked.

#### Exploring Being Autistic Programme

The intended outcomes of *Exploring Being Autistic* were to enable participants to: develop a good understanding of what autism means to them and to identify a path forward; experience a connection with a peer group and decrease anxiety; be better able to build on autistic strengths and mitigate autistic challenges; and be better able to explain their condition to others, request appropriate accommodations, and adapt some of their own behavior. Designed and led by an autistic facilitator (CH), the sessions comprised information about autism as well as (optional) role play and discussion (see Table 2 for further details).

**Table 2:** Overview of exploring being autistic

Week	Topic	Content
1	Introduction and establishing ground rules	The structure of the group. Overview of contents. Introductions. Sharing autism histories
2	Diagnosis/Identification of Autism—what does it mean for you?	Initial feelings about autism. Is the label limiting or liberating? Disclosure in different contexts
3	Social communication and Theory of Mind	Factual vs social communication. Implicit and explicit rules. Non-verbal communication
4	Improving social communication	Locating and moderating emotions. Face theory. Empathy. Social skills vs social connection
5	Sensory issues	Stimming. Hyper and hypo sensitivities. Emotional sensitivity
6	Executive dysfunction	What gets in the way of moving from motivation to action? Strategies. Time keeping
7	Attention and disparate ability profiles	Attention shifting, mono attention, special interests. Spiky ability profiles
8	Flexibility	Dealing with change. Use of routines. Perfectionism. Boundaries
9	Anxiety, depression and mental health	The relationship between autism and mental health. Benefits and pitfalls of disclosure
10	Where to from here?	Consolidation. Autism and the law. Planning for the future. Course evaluation

#### Post-programme Interviews

Participants were invited to take part in an interview with one of the researchers (LC) at the end of the programme, and again 6 months later. To ensure that the participants felt comfortable taking part in an interview with the researcher (who does not identify as autistic), the researcher was invited to the final session of the programme to meet and spend time with the participants. At the request of the facilitator (CH), the researcher discussed her background and interest in autism research, and she spoke about her current research interests. Participants were also given the opportunity to ask questions about the researcher’s work generally, as well as the current evaluation. This process was essential: establishing trust and a reciprocal relationship for collaborative, inclusive research.

Interview protocols, developed for the purpose of this study, were used to guide discussions. All interviews began with a rapport building phase, in which the interviewer provided the interviewee with details on what to expect and the purpose of the interview.

The first set of interviews, conducted immediately after the programme ended, covered the following topics: previous attendance at support groups (probing for positive and negative aspects of these groups); motivations for joining the group and what they had hoped to gain; whether the programme met their expectations; and their overall appraisal of the programme (what worked well, and what could have been improved). Interviews concluded with a discussion of the autistic-led nature of the programme, and participants were offered the opportunity to add any further thoughts or ask any questions.

The second set of interviews, conducted 6 months after the programme ended, focused on participants’ reflections on the programme (with the benefit of hindsight). The following topics were covered: whether the participant was pleased that they took part in the programme; if/how they felt the programme affected them; aspects of the programme that they thought were helpful and unhelpful; potential content or topics of discussion that the programme could have usefully covered; and whether participants had attended any support groups since the programme ended. As a final discussion point, the interviewer asked whether the participant had kept in contact with any members of the group and whether they had utilised the suggested social networking platform to assist them in doing so.

All questions were open ended, in order to allow interviewees to provide their honest views without influence from the interviewer. Interviews were conducted either face-to-face or by telephone, according to logistics and participants' preferences. The mean length of the interviews was: 27.59 min (SD = 13.58, range 9–51) for the initial follow-up interviews and 16.19 min (SD = 6.03, range 5–24 min) for the 6-month follow-up interviews.

### Data Analysis

Interview data (from both time points) were transcribed verbatim and analysed in line with Braun and Clarke’s (2006) essentialist framework for thematic analysis. Throughout, participants’ names have been replaced by pseudonyms. The author who conducted the interviews (LC) led the analyses. This took an iterative approach, conducted alongside data collection. The order in which interviews were conducted and analysed was as follows: (1) initial interviews for participants in iteration one; (2) initial interviews for participants in iteration two; (3) 6 month follow-up interviews for participants in iteration one; and (4) 6 month follow-up interviews for participants in iteration two. Following round one of data collection, LC independently familiarised herself with the transcripts, reviewed the semantic content of the data and produced preliminary codes and themes without a pre-existing coding scheme. A similar approach was taken following each subsequent round of data collection; assimilating the new data into the original codes and themes, and amending the codes and themes where necessary. [Note that, as the codes and themes from participants in iterations one and two overlapped considerably, the decision was made to present data from the two iterations as one homogenous group.] To enhance the reliability of the thematic analysis, two additional researchers (MA and JD) subsequently familiarised themselves with all interview data (across both iterations of the programme, and both time points). Using the first author’s codes and themes as a framework, the team discussed the coded data, reviewed discrepancies and decided on final themes for the interviews. Pre-programme questionnaire responses were analysed by two researchers (MA and JD). They followed the same process outlined above, including coding and discussion, to identify themes relating to motivations for attending the programme.

## Results

### Motivations for Attending the Programme

Pre-programme questionnaires were used to better understand participants’ motivations for taking part in the programme. Responses were organised into three themes: (1) exploration of autism; (2) empowerment; and (3) developing practical strategies and coping mechanisms.

#### Reason 1: Exploration of Autism

Participants explained how they engaged with the programme to gain “a deeper understanding of autism” (Harry); generally, but also in relation to themselves: “[I hope to gain increased] self-awareness and self-knowledge” (Brooke). They noted that they wanted to consolidate existing knowledge: “I have done some reading and attended conferences, but in a piecemeal way, about different aspects of autism, and I hope the course will pull the information together” (Fiona); as well as gain a better understanding of specific aspects of autism: “[I want to] understand more about … how my anxieties led to depression and suicidal thoughts” (Kayla). For some participants, exploration was needed to investigate whether they felt that they met the criteria for an autism diagnosis and, if so, whether it would be worthwhile to proceed with a formal diagnostic assessment: “[I wanted] to investigate if self-identification would be a better option for me than trying to obtain a formal assessment/diagnosis from the NHS” (Danielle). Other participants wanted to explore disclosure of autism, to “better understand who, when and how to disclose that I’m autistic. In particular I’m interested in disclosure to family and future employers” (Grace).

#### Reason 2: Empowerment

A common motivating factor for participating in the programme was based upon “empowerment” (Nigel) and a desire to feel accepted: “I'm looking to meet other like-minded women with a late diagnosis who have struggled for most of their lives but without knowing why” (Emily). Participants wanted to “meet others like me” (Callum), gain “confidence” (Brooke) and “feel less isolated” (Fiona). Linked to this was a desire to “explore the positive aspects of autism” (Andrew).

#### Reason 3: Developing Practical Strategies and Coping Mechanisms

Participants wanted to learn from one another: “hopefully I will learn something from [the other group members]” (Danielle). Specifically, they wanted to be able to “develop strategies of how to support myself and others like me” (Callum). They also wanted the programme to help them “work through mixed emotions” (Fiona) following diagnosis, and assist them in navigating challenging experiences (e.g., transitions).

In the pre-programme questionnaire, participants were also asked whether they received enough information prior to attending the programme. Encouragingly, all said that they felt that they did receive enough information, with one commenting that this was particularly due to the helpful one-to-one meeting with the facilitator, which took place prior to the start of the programme.

### Evaluation of the *Exploring Being Autistic* Programme

Three key themes were identified from the interview data at both time points (both immediately after the end of the programme, and again 6 months later): (1) Appreciation of the autistic-led nature of the programme; (2) Unity in diversity; and (3) Developing a positive, practical outlook on autism.

#### Theme 1: Appreciation of the Autistic-Led Nature of the Programme

Some participants were not aware that the programme was organised and led by an autistic person when they initially signed-up, and only in hindsight realised the benefits: “I don’t think that necessarily would have occurred to me before, but now that I’ve done the group and have been led by [the facilitator], who I know is autistic, I liked that and that worked for me” (Brooke). Others reported that they would have engaged with a group led by a neurotypical person, but expressed a preference for the group facilitator to be autistic: “it’s one of the strengths of the course in that you do have that kind of perspective from the person facilitating… it contributed to a feeling of a shared safe space, and of a lessening of judgment … I would engage [with a group led by a non-autistic person] but I would always prefer to be with a person on the spectrum” (Callum). For others, the autistic-led nature of the programme was a key reason for attending:

“I don’t think I would have been quite so keen to come and have [a non-autistic person] lecture me … [the autistic facilitator] made me feel like she was much more understanding and you could open up more, and you could be really honest…I don’t think I would have done that if it was someone who didn’t have the personal understanding and experience of being autistic themselves … to me that was really important and I probably wouldn’t have come had it not been [led by an autistic person]” (Danielle).

The autistic-led nature of this programme was reported to be a welcome contrast from less positive previous experiences with neurotypical professionals in the past:

“I was diagnosed by two professionals who weren’t autistic themselves and I felt really scrutinised, I felt so vulnerable … judged. I was being watched by two people for a couple of hours and I found that quite intimidating … I accepted it as part of the process by its nature really, you’re being diagnosed, but it didn’t feel very friendly, a bit soul destroying” (Brooke).

It was also felt that the autistic facilitator was, perhaps, more qualified than a neurotypical person to lead such a course: “if she has autism herself, then she understands about autism…[an autistic person would] know how to present it better—they understand—whilst sometimes people who don’t have autism don’t understand” (Isabelle). Related to this, the perceived qualities of an autistic facilitator were felt to lend themselves well to leading an autistic group:

“if you have a non-autistic person running the group, they are perhaps going to be less tolerant and less patient of the way that some of us can go off on a tangent, but for us the tangent is just as important as the subject matter we were originally talking about” (Kayla).

One participant commented that:

“we are reaching a stage where we need autistic-led information, groups, support groups and workshops because, particularly with adults, it just doesn’t work any other way. There’s a certain feeling of imposition if someone is not on the same wavelength as you, so I think it’s important that [autistic-led groups] happen and just keep going forward” (Jane).

Not many participants had experience of attending a support programme led by neurotypical professionals with which to compare this programme. Those who did have experience of non-autistic led groups referred to them as being too formal and structured: “we’d get into a discussion and then it’d be like ‘no, we’ve got to pull it back to tick this box’ and so on” (Jane). However, the positive aspects of previously attended non-autistic-led programmes were noted: “it was a good experience to be with other people and be able to see other people’s experience” (Jane). Many reported simply being pleased that something was available: “it was good that there was something rather than just leaving you with the diagnosis and running away” (Nigel). It was also questioned whether there needed to be a dichotomy between autistic-led and non-autistic-led programmes: “I think there should be a collaboration…I think there’s more positive outcomes from collaboration and openness, than from exclusion” (Lucy).

The fact the facilitator was autistic also conferred a benefit in the sense that the facilitator was felt to have a very positive view of what it was to be autistic: “I think she’s a very positive role model” (Fiona). This changed participants’ perceptions on what it meant to be autistic:

“it’s not this really negative, awful position being me. I’m different…I have got a lot of strengths, they’re just intrinsic as being part of me, it’s who I am and it’s partly because I am autistic, so the group made me aware more of myself in a positive way” (Brooke).

Indeed, other participants also reported important attitudinal changes in what it meant to be autistic:

“I thought [being autistic] is not going to change who I am, and I think I didn’t feel like that before I came to the programme” (Danielle).

Whilst some participants felt that the facilitator being autistic “didn’t get in the way of anything” (Harry), others noted that it did occasionally impact on the delivery of the material: “occasionally I found her delivery a bit disjointed, and she once forgot some of the teaching material” (Fiona). However, this was not perceived negatively: “neither mattered much to me and may even have added an element of 'shared vulnerability’” (Fiona). Equally, it was felt that whilst having an autistic facilitator was helpful, it was actually the mix of the facilitator’s personal and professional knowledge that was key. Whilst the facilitator “didn’t often talk about her own experience” (Fiona), it did “seem appropriate” for her not to do this and “she struck just the right balance between being professional as a group facilitator, and participating as a fellow autistic” (Fiona). Ultimately, it was the facilitator’s professional expertise that seemed to be particularly important: “her facilitation skills were lovely, and very skillful” (Grace); “she’s really good in keeping control and keeping things moving at a pace that made sure that we had time to explore ideas but we also had to come back to…a certain routine. That was really important” (Emily). It was suggested that it may be helpful, in future, to have two facilitators, due to the sometimes “emotionally draining” (Paula) content being discussed within the group. Participants questioned: “if people did ever get really upset or distressed, who would carry on with the group? Who would manage that?” (Grace). Indeed, greater screening of mood was suggested as important in future iterations of the programme.

#### Theme 2: Unity in Diversity

Participants commented positively on the diversity within the groups:

“the stereotype of what [autism] is, is not true, and we are actually all very different and some of us, actually, are quite extrovert and like being around people. Our jobs, our interests, and our sort of histories are very different. Some of us had children and young people, some not; some have been married; and, again, the age difference, some people in their 20s and even people in their 60s, so there was a whole range” (Grace).

“I like the fact it’s mixed, I like the fact it’s male and female, I like the fact there’s a mix of age groups because there’s some younger people and some older people—I think that’s really key…you need to see it in all areas, because that’s the nature of the whole thing itself—it doesn’t just pick. If you had ten people like me in a room, we wouldn’t learn anything…I think that was very important” (Emily).

This diversity was perceived as particularly positive for those who previously had limited experience of meeting other autistic people: “[each group member] seemed to be the kind of person you’d meet any day and not realise they were on the spectrum, so that was a surprise” (Andrew); “meeting the group was massively relevant for me, because it’s one thing reading about it on the Internet but when you’re sitting in a room…that’s an epiphany” (Emily).

In addition to the mixed demographics, participants were at very different stages in their diagnostic journeys—some had received their autism diagnosis some time ago, others had received their diagnosis quite recently, whilst others self-identified as autistic (often debating whether to pursue a formal diagnosis): “I think it was really good that we had people at different stages” (Danielle); “we’re all at different stages…two [participants in the group] were very recently diagnosed [and] were quite different in some ways from the other members of the group” (Grace).

Despite the diversity of the group, participants overwhelmingly reported a sense of belonging: “just talking to each other about our experiences was to me the strong point…you gain from that, you feel legitimised by other people having the same experiences, so it means you’re not just one weird outpost” (Callum). Participants reported feeling comfortable and connected with one another:

“the level of being comfortable with everybody was quite high, so that was nice. It didn’t feel like you couldn’t say something out loud, which was helpful cause I do a lot of self-editing—if I don’t feel confident that there’s not going to be gasps of horror or strange looks, then I just won’t say anything—so that was particularly helpful” (Jane).

Participants reported that they did not always feel this sense of belonging in non-autistic groups: “finding people that have been through similar experiences to myself…my friends and family could never understand why I had so much difficulty, whereas the people in the group completely understood” (Kayla); “it was like being in a nursery with no fighting for the first time. It was like finding the classroom where you could actually be normal and make friends and I don’t think any of us have experienced that” (Paula). However, participants also reported that they had not felt this with some autistic groups, especially when previous groups (unlike the current group) represented what was perceived to be a very wide range of autistic individuals:

“if the way that your iteration of the spectrum manifests itself is not as extreme as other people, it can be difficult to share experiences and similarities, it can be difficult to note your place there and where you fit in and that can be isolating too…you go somewhere like [an autistic event] and it’s nice to have a space and it’s refreshing but the feeling that you can get if you don’t meet people that seem to be similar to you is a second, not rejection, but distance” (Callum).

This sense of belonging was felt to be especially important given that the participants were “somewhat socially isolated” (Nigel) and/or had difficulties with friends and family not really understanding them or their difficulties:

“my friends and family could never understand, why I had so much difficulty, whereas the people in the group completely understood. I think not only does the content of the actual course itself [confer a benefit in terms of] the understanding that came with that, but also knowing that other people were going on that journey with me. It made it a lot easier and I’ve come out from it feeling so much lighter than I’d been since my early teens. It’s been- sorry, I’m going to get emotional now—[pauses to cry]—it’s been absolutely life-changing” (Kayla).

The programme was also reported to “open up a whole new social world, which has been fantastic” (Kayla); and enabled participants to “develop a new community around autism being a common feature” (Olivia). Participants who reported that they had struggled or experienced a lot of challenges in their everyday lives enjoyed the opportunities to interact with other autistic people who were perceived as being successful:

“it’s inspirational when you meet autistic people who are higher functioning than you…one of the other members in the group has got quite a good job in finance and she holds down that job and I’m unemployed at the moment and I found that inspirational—if she can do that, then I can do that” (Nigel).

This sense of belonging appeared to be linked to the process of sharing personal experiences, which had a number of benefits to the group, particularly in relation to self-awareness: “I had so many lightbulb moments, I thought oh my god, I did that, oh my god! It was just so uncanny. It was really, really amazing to become aware of those things” (Brooke). This was also reported to enable participants to frame difficulties in a more positive way: “[I had] a couple of lightbulb moments about things I’ve suffered from for years and then realising it’s just a symptom of a neurological condition and not something that is innately wrong with your character, that makes it a lot easier” (Nigel). This sharing of personal experiences was, in some ways, felt to be even more important than the structured autism knowledge they were receiving:

“peer to peer, and people are talking to each other about their experiences, I think that’s really helpful … you need a facilitator but I really like it when there was more just people talking together amongst themselves and I know that [the facilitator] did try to do that as much as possible, I think that’s very much a feeling that she wanted from the group … she obviously wanted to communicate a situation but she also wanted people to interact with each other and that, for me, was a really strong part” (Callum).

Concern was, however, raised about who should be eligible to complete the course; more for future iterations than in relation to the current cohorts:

“from my experience, maybe even if you’re within a year, or yeah 6 months of your child being diagnosed perhaps, you know, perhaps it’s not the best time of doing it, because you’re trying to process that and then your own stuff” (Danielle).

#### Theme 3: Developing a Positive and Practical Outlook on Autism

Participants reported that the programme improved their outlook on autism, and this made a real difference in their day-to-day life: “since I’ve been coming to the group there’s been a marked difference in how I am and how I see the world” (Kayla). This newfound outlook was reflected in a number of areas of their lives, and was also related to the educational aspect of the programme: “[It’s helped me to] understand some of the challenges that I face and why I do, um, face those challenges” (Danielle). This knowledge enabled participants to speak about autism with others: “I could then start to talk about what is autism, what are autistic people like, what are their strengths, … facets of the autistic community and also what are the challenges people have” (Olivia). Increased understanding about autism also gave participants “a much broader interest [in autism]” (Andrew), and encouraged some to develop their knowledge further: “I have also gone onto reading a couple of books that I found very good” (Lucy).

The course content was felt to be important for participants struggling with their diagnosis of autism:

“Becoming aware that I had Asperger’s, it kind of made me focus on my weaknesses … [the facilitator] presented a very full view of being on the spectrum, which included a lot of really positive stuff as well and strengths … [this] gave me a more rounded picture of being on the spectrum and made me feel that actually, there were a lot of positive things that I had that I could focus on” (Brooke).

The comprehensive and positive representation of autism that the programme promoted empowered people to “accept that I was a part of this” (Isabelle). Acceptance was often coupled with increased self-awareness: “It really made me aware of things about myself that I hadn’t even been aware of before… it’s only through that group talking that you can really see things and accept it and laugh about it” (Emily);

“It was a case of self-discovery, to be honest … to actually go through the programme and go, oh my god, this is why that’s happened and this is why I’m like this, it has just changed my whole outlook on life and to the point where I can make sense of things now.” (Kayla).

Being more self-aware meant participants were able to realise their own behaviours as they were happening, draw on their improved knowledge to explain why they might be doing or feeling something, and then search for informed solutions to problematic situations:

“I’m in a situation and something arises that I catch myself and go, oh yeah, this is the bit that I understand now,… Whereas before I wouldn’t understand it and I might… [have] got really angry or depressed or really anxious… [now] I can sort of see what’s happening and find a way out of it.” (Emily).

There was also felt to be an extended impact beyond those that attended the programme. For example, one participant reported that: “[the programme] gave me a different way of thinking about autism both for myself and also for my children” (Danielle).

Learning and talking about autism in a positive way led to participants gaining a positive, practical outlook on autism. This ranged from general attitude changes to applying more specific strategies to tackle challenges encountered in day-to-day life. One example of a broad attitude change was related to increased self-awareness:

“I used to… put so many demands on myself to be at a really high level about everything but now I kind of get that I’m not going to be able to do that because I haven’t got the capacity to do that.” (Emily).

Another participant changed her approach to how she presented herself following discussions around social camouflaging:

“there’s one particular thing that stuck in my mind that we discussed about being authentic… just because you might come across as a bit weird, it doesn’t mean people… won’t like you… people can tell if you’re being sort of fake… so I’ve kind of done less masking.” (Grace).

Other participants were satisfied that they could deal with their diagnosis more practically: “this programme had really prepared me and I really dealt with some of those kind of issues about thinking about who I was going to tell, who I wasn’t going to tell” (Danielle).

Many of the more specific practical solutions were reported to be associated with combating mental health problems, particularly anxiety:

“One week where I was feeling ‘off’, I remember going home and thought about [the session] and did something constructive about it for once, rather than spend my time worrying, and it was really useful…we were changing rooms at home and I was feeling completely wrong…I realised that it was probably this change…and I managed to do something to remove some of the stress that was going on” (Jane).

The positive practical outlook was also demonstrated by participants feeling better equipped to address anxiety “I’m living by the moment now… the anxiety hasn’t disappeared, it’s still there but I understand the reasoning behind it, so I feel it makes me better able to cope with the day-to-day things that used to cause me problems” (Olivia). Changes in outlook were also reported to have an impact on what participants were able to do:

“going places when I haven’t been there before, I used to be a bundle of nerves and to the point where sometimes I’d actually not go so it would actually stop me from doing things, whereas now I feel like I’ve got it under control… I understand the reasoning for the anxiety, which means I’m braver than I used to be and I’m more likely to try things because actually I know the anxiety is to do with my autism and not actually because it’s anything to worry about.” (Olivia).

Additional practical solutions were related to addressing sensory issues in the workplace:

“[I got given] some tools where I can go into work and say ‘you have your music really loud, it makes it really hard to work in an open plan office’. I’m not going to say [it’s] because I’m autistic, but [the facilitator has helped me] word it in a way that gives myself a little bit of power to say ‘I’m gonna need this or that or whatever’, so practical strategies are really helpful” (Emily).

Additionally, participants reported an improved confidence with social issues such as eye-contact:

“although I’m quite comfortable with eye contact with people that I know, before when I was out and about, I tended to look at the pavement, look at the floor and not particularly look at people, whereas now where I’ve changed… I’m more confident, happy… I walk with my head up, I look at people.” (Olivia).

Practical solutions were deemed so helpful that participants reflected that they would like to do a follow-on course that provided even more practical information:

“it would be great if you, again, in 6 months’ time, had level two and then people who had done this course could go on to level two and it could be more about living with the diagnosis, giving maybe support, but practical stuff about how to cope with people in your life” (Emily).

There was a longing for some form of “continuation space” (Callum), as participants valued “having a space to allow the information to sink in” (Emily). Indeed, some participants noted anxiety over the programme and its associated support ending: “it’s a really supportive, kind, non-judgmental, environment… you’ve just built that up and then it’s cut off” (Grace). The same participant went on to describe that after a support group “there’s no one really, there’s nothing really there. It’s just, you’re just on your own really” (Grace). Some did note that there were, however, some options available to them:

“now the course has ended, I feel quite upset about losing the support I was getting through attending the group…I also know that [the facilitator] runs once a month groups that I can attend, so it’s not as if I’m being cast adrift…that is a positive thing” (Brooke).

Many questions were raised about how further support options could be organised. For example, it was noted that it was difficult to decide when to raise the possibility of some form of continuation space: “I think it would be good to mention it at the beginning but I think people won’t know at that time whether they want to continue” (Callum). The facilitator had suggested an online forum, but although participants were receptive to the idea of maintaining contact with the group, there was discussion about the appropriability of maintaining contact online: “there was an Internet forum, that [the facilitator] was talking about setting up, and that’s great. I think that would be nice for the group themselves” (Callum); “it needs to be fairly restricted in numbers otherwise there’s going to be lots of different conversations going on at once” (Andrew); “[there is a] question of confidentiality and anonymity” (Andrew). Another noted that “the Internet forum is a happy medium but it would be good if people continued meeting up but there are financial restrictions” (Callum).

Follow up interviews 6 months later revealed that the programme did present participants with an opportunity to meet peers with whom they could keep in touch with, and some did organise and initiate such meet-ups: “it’s nice to not just go to a group and find out about stuff but to be able to build up more support structures” (Olivia). This opportunity also extended to other autistic peers that did not attend the programme:

“it was a shame it was coming to an end and I was thinking, well, why don’t we start meeting up more and getting other people on the spectrum in the local area who feel they don’t have the support they need at the moment to join in as well” (Olivia).

This group was warmly received by those who did attend: “It’s nice to have that group because, because we’re all very familiar, we all understand each other… it’s good to have that network post-group, which has been fantastic.” (Kayla). Other participants confirmed that they did keep in touch online: “I know quite a few of us use [social media], which is actually how I’ve really stayed in touch with one of the group members.” (Danielle). Selecting an appropriate online platform to facilitate keeping in touch seemed to be key:

“I can’t say I found [the online group] that useful, to be honest, just because I think a lot of people weren’t using it and because… of the format… I don’t think a lot of people use [the platform] in their everyday life… [so] I don’t think people really, really did it.” (Grace).

Participants expressed a wish for other autistic adults to benefit from the programme in the same way that they did: “it would be great if this expanded and more people had options to do it in more places” (Callum); “It has been amazing and I think there were a lot of people who would really benefit from coming on it and I think it would be a real shame if we couldn’t carry on with them” (Danielle).

## Discussion

This initial evaluation demonstrated that an autistic-led peer support programme for autistic adults (either formally diagnosed or self-identified as autistic) was well-received, with participants benefiting greatly, in many different ways. Specifically, participants were positive about the autistic-led nature of the programme, developed a sense of unity within the diverse group of attendees, and were able to use their experiences to foster a positive, practical outlook on autism. Whilst participants were complementary about the skills and expertise of the facilitator, it was encouraging that many of the positive aspects noted by participants were in relation to both the structure and general principles of the programme (e.g., the positive nature of the syllabus, the diversity of group members); all of which could be taken forward by other (trained) autistic facilitators in the future.

The autistic-led nature of the programme was seen as a particularly positive aspect of the programme. This raises two key points for discussion: first, whether all support programmes should be autistic-led; and second, whether professionals working with autistic people need to disclose their neurology (i.e., whether they themselves are autistic or not). Recent research has demonstrated poorer performance and lower rapport amongst groups of mixed neurology (i.e., autistic and neurotypical participants) relative to groups of the same neurology (i.e., all autistic, or all neurotypical, participants) (Crompton et al. 2019; Heasman & Gillepsie, 2019). Whilst this does not suggest that autistic and non-autistic people cannot work together, it does suggest a benefit for autistic professionals working with autistic groups. This may be particularly relevant given the nature of the current programme: to provide an analogy with another minority group, one might query the validity of men teaching women about women’s issues, but it is widely accepted (and even commonplace) for non-autistic people to provide education and support about autism to autistic people. Notably, a researcher who does not identify as autistic conducted interviews for this evaluation, and participants reported this experience to be positive. For example, one participant noted: “I have taken part in other surveys and other research, for completely different things, and yours was probably one of the clearest ones I’ve ever seen.” (Katherine); and another remarked positively: “I didn’t feel intruded upon in any way” (Callum). This is likely to, at least in part, relate to the efforts made by the researcher (on the advice of the facilitator) to engage, and develop a trusting relationship, with the participants prior to the interview—somewhat levelling the power balance. As with other research involving autistic people, it seems that—irrespective of the neurology of participants and professionals—building trusting relationships, based on mutual respect, is key (e.g., Crane et al. 2019a, b; Fletcher-Watson et al. 2019). A critical lesson for future programmes is the importance that should be placed on co-production and cultivating respectful, trusting relationships with group members.

The diverse nature of the group was well-received by participants, particularly as this helped them to learn more about the nature of autism. It should be noted that, whilst the group did comment on the diversity of participants, the groups were relatively homogenous in that they all comprised autistic adults who were able to participate in a social peer group setting. Questions are often raised about which groups of autistic people are most worthy of support and research attention, with calls for greater attention to be given to autistic people with co-occurring intellectual disabilities (e.g., Russell et al. 2019). Yet it is important to move away from discussions surrounding which groups are most worthy of support towards an acknowledgement that we should be fighting for access to high quality support for *all* autistic people. This is particularly pertinent for participants who would be eligible for the *Exploring Being Autistic* programme. Participants noted how the group included people that they would not have “realised were on the spectrum” (Andrew). For this reason, the *Exploring Being Autistic* programme was perceived as different to groups participants had previously attended, because they met people “similar” to them, with “less extreme” presentations of autism. This highlights one of the many pervasive misconceptions about autism, such as the mistaken belief that autism always co-occurs with intellectual disability (Gillespie-Lynch et al. 2015). Autistic adults who are verbally and cognitively able are perhaps at greatest risk of having their support needs overlooked, as they tend to be seen as “too ‘normal’ to be different and, equally, too ‘different’ to be ‘normal’” (Crane et al. 2019a, b, p. 484). The result of such misconceptions is that those who do not fit these more familiar presentations of autism tend to ‘fall through the cracks’ (Crane et al. 2019a, b, p. 484). While *Exploring Being Autistic* was unique in recognising the situation of many autistic adults who were not provided for by most support services, this is not to say that the participants were actually unusual or rare in their presentation of autism, or that they were less in need of support. A key recommendation from the current work is for there to be greater recognition of the need to provide role models, programmes and peer-support catering specifically for autistic individuals that are verbal and cognitively able; a group who have a range of very distinct—but, unfortunately, often negative—outcomes (e.g., Ayres et al. 2018; Hirvikoski et al. 2016; Moss et al. 2015; Orsmond et al. 2013; Shattuck et al., 2016).

Encouragingly, the *Exploring Being Autistic* programme provided participants with a positive, practical outlook on autism. The sharing of experiences was reported to be particularly useful in this regard. Autistic adults often internalise negative social experiences, leading to mental health problems (Crane et al. 2019a, b). Yet hearing the stories of peers with similar experiences (and sometimes learning about how they overcome their challenges, and even adopting such strategies themselves) provided a sense of empowerment. For autistic adults who are unable to attend a peer group setting, or for those lacking programmes such as *Exploring Being Autistic* in their locality, written accounts from autistic adults (e.g., Hearst 2019) may serve a similar purpose. This may be particularly crucial for autistic adults immediately post-diagnosis, as the diagnostic process itself often has a focus on negative, rather than positive, aspects of autism (Crane et al. 2018). Future programmes should continue to ensure that there are opportunities for group members to share their experiences, as well as discuss strategies to address challenges they face.

Whilst the experience of participating in the group was reported to be positive, participants did express their dismay about the end of the programme (at week ten) and the lack of follow-on support. Members of the autism community (including autistic adults, but also parents of autistic children) often report feeling ‘dumped’ and ‘directionless’ after an autism diagnosis (Crane et al. 2018, p. 3767), and it is important that the same feelings are also avoided after the provision of programmes such as *Exploring Being Autistic*. This echoes the findings of other research; for example, young autistic adults have reflected on the benefits of proactive, open-ended mental health support provided by Child and Adolescent Mental Health Services in the United Kingdom, yet have lamented the reactive nature of mental health support adopted by Adult Mental Health Services (who do not provide ongoing support and tend to admit patients only when in mental health crisis) (Crane et al. 2019a, b). Given funding constraints, open-ended and long-term support programmes for autistic adults are unlikely to be made widely available. Attention therefore needs to be given to how to make short-term support for autistic people more sustainable. Whilst those who took part in *Exploring Being Autistic* were encouraged to attend ongoing monthly peer support (as part of a broader ‘drop-in’ group) and take the initiative in maintaining the relationships developed through the programme, this was with limited success: for example, some participants stayed in touch, but this was rather inconsistent. A key learning outcome from this programme, which will be important to address in similar future programmes, is the need to dedicate additional time to discuss strategies for ongoing engagement with an autistic community the end of the programme.

Finally, it is important to consider the strengths and limitations of this research. A key strength of this evaluation was the use of in-depth, qualitative interviews to provide detailed insights into participants’ views and experiences of the *Exploring Being Autistic* programme; providing an evidence base upon which future peer support programmes can be based. A further strength was the use of participatory research principles (e.g., Fletcher-Watson et al. 2019) to facilitate genuine, co-produced research. Specifically, an autistic facilitator (CH) devised and led the *Exploring Being Autistic* programme, and co-designed the research to ensure the evaluation was accessible and acceptable for participants. In terms of limitations: First, this was a small scale, initial evaluation and conclusions should be tentative. Larger scale evaluations of similar programmes are needed, to provide further evidence for the characteristics of effective post-diagnostic support programmes for autistic adults. Second, it would be difficult to reliably replicate an evaluation of *Exploring Being Autistic* as the programme is not currently manualised. However, it should be noted that the goal of the project was to learn more about autistic-led peer support as an approach, rather than to provide an evidence-base for this specific programme. It should also be noted that this course has now run a number of further iterations, whereby the content has been refined, there is an assistant facilitator and there are plans to put the programme online. Third, it was difficult to determine whether the success of the course was attributable to the skills of the facilitator or to the content, structure and nature of the programme itself. However, the structure and general principles of the programme were discussed favourably, suggesting these could be taken forward by other, trained autistic facilitators in future. Finally, as the facilitator was involved in devising and promoting the programme evaluation (although not involved in the qualitative analysis), participants may have been positively biased in their feedback. Having said this, the interviews were conducted by an independent researcher and participants did remark on weaknesses of *Exploring Being Autistic*, which suggests they did give a reliable assessment of the programme.

In conclusion, this research represents a successful initial evaluation of an autistic-led post identification/diagnosis peer support programme. Participants were motivated to attend the programme for several reasons: exploration of autism; empowerment; and the development of practical coping strategies. Three key themes were identified from post-programme interviews, which revealed an appreciation of the autistic-led nature of the programme, a sense of unity within the diverse group of participants, and the development of a positive and practical outlook on autism. This initial evaluation provides important insight into aspects that could be useful for future programmes, which could in turn generate a larger evidence base about post-diagnostic peer-support groups. Based on the current preliminary evidence, recommendations for future programmes include: ensuring that peer-support programmes are autistic led; accepting a range of group members, including those that self-identify as autistic; providing material on practical tips and solutions to challenges group members face; and enabling ongoing support after the programme finishes.

## Footnotes

1 Note that ‘high functioning’ autism has never been a formal diagnostic label and there is growing recognition of the fallacy in referring to individuals as having ‘high’ or ‘low’ functioning autism (see, for example, Alvares et al. 2019).

2 There is debate about how autism is—and should be—described. In this article, we use ‘identity-first’ language (i.e. ‘autistic person’) rather than person-first language (i.e. ‘person with autism’), in accordance with the views of autistic activists (e.g. Sinclair 1999) and many autistic people and their families in the United Kingdom (Kenny et al. 2016).

## References

[CR1] Alvares GA, Bebbington K, Cleary D, Evans K, Glasson EJ, Maybery MT, Pillar S, Uljarević M, Varcin K, Wray J, Whitehouse AJ (2019). The misnomer of ‘high functioning autism’: Intelligence is an imprecise predictor of functional abilities at diagnosis. Autism.

[CR2] Ayres M, Parr JR, Rodgers J, Mason D, Avery L, Flynn D (2018). A systematic review of quality of life of adults on the autism spectrum. Autism.

[CR3] Bargiela S, Steward R, Mandy W (2016). The experiences of late-diagnosed women with autism spectrum conditions: An investigation of the female autism phenotype. Journal of Autism and Developmental Disorders.

[CR4] Braun V, Clarke V (2006). Using thematic analysis in psychology. Qualitative Research in Psychology..

[CR5] Crane L, Adams F, Harper G, Welch J, Pellicano E (2019). ‘Something needs to change’: Mental health experiences of young autistic adults in England. Autism..

[CR6] Crane L, Batty R, Adeyinka H, Goddard L, Henry LA, Hill EL (2018). Autism diagnosis in the United Kingdom: Perspectives of autistic adults, parents and professionals. Journal of Autism and Developmental Disorders..

[CR7] Crane L, Chester J, Goddard L, Henry LA, Hill EL (2016). Experiences of autism diagnosis: A survey of over 1000 parents in the United Kingdom. Autism..

[CR8] Crane L, Davidson I, Prosser R, Pellicano E (2019). Understanding psychiatrists' knowledge, attitudes and experiences in identifying and supporting their patients on the autism spectrum: online survey. BJPsych Open.

[CR9] Crompton, C. J., Fletcher-Watson, S., & Ropar, D. (2019). Autistic peer to peer information transfer is highly effective. https://doi.org/10.31219/osf.io/j4knx. 10.1177/1362361320919286 PMC7545656 32431157

[CR10] Dawson-Squibb J-J, Davids EL, de Vries PJ (2019). Scoping the evidence for EarlyBird and EarlyBird Plus, two United Kingdom-developed parent education training programmes for autism spectrum disorder. Autism..

[CR11] Fletcher-Watson S, Adams J, Brook K, Charman T, Crane L, Cusack J (2019). Making the future together: Shaping autism research through meaningful participation. Autism..

[CR12] Fletcher-Watson S, Happé F (2019). Autism: A new introduction to psychological theory and current debate.

[CR13] Gillespie-Lynch K, Brooks PJ, Someki F, Obeid R, Shane-Simpson C, Kapp SK (2015). Changing college students’ conceptions of autism: An online training to increase knowledge and decrease stigma. Journal of Autism and Developmental Disorders.

[CR14] Gordon K, Murin M, Baykaner O, Roughan L, Livermore-Hardy V, Skuse D, Mandy W (2015). A randomised controlled trial of PEGASUS, a psychoeducational programme for young people with high-functioning autism spectrum disorder. Journal of Child Psychology and Psychiatry..

[CR15] Gould J, Ashton-Smith J (2011). Missed diagnosis or misdiagnosis? Girls and women on the autism spectrum. Good Autism Practice (GAP).

[CR16] Hansen SN, Schendel DE, Parner ET (2015). Explaining the increase in the prevalence of autism spectrum disorders: The proportion attributable to changes in reporting practices. JAMA Pediatrics..

[CR17] Happé FG, Mansour H, Barrett P, Brown T, Abbott P, Charlton RA (2016). Demographic and cognitive profile of individuals seeking a diagnosis of autism spectrum disorder in adulthood. Journal of Autism and Developmental Disorders..

[CR18] Hearst C (2019). Being Autistic: Nine adults share their journeys from discovery to acceptance.

[CR19] Heasman B, Gillespie A (2019). Neurodivergent intersubjectivity: Distinctive features of how autistic people create shared understanding. Autism..

[CR20] Hirvikoski T, Mittendorfer-Rutz E, Boman M, Larsson H, Lichtenstein P, Bölte S (2016). Premature mortality in autism spectrum disorder. The British Journal of Psychiatry.

[CR21] Jones L, Goddard L, Hill EL, Henry LA, Crane L (2014). Experiences of receiving a diagnosis of autism spectrum disorder: A survey of adults in the United Kingdom. Journal of Autism and Developmental Disorders..

[CR22] Kanner L (1943). Autistic disturbances of affective contact. Nervous Child: Journal of Psychopathology, Psychotherapy, Mental Hygiene, and Guidance of the Child.

[CR23] Kenny L, Hattersley C, Molins B, Buckley C, Povey C, Pellicano E (2016). Which terms should be used to describe autism? Perspectives from the UK autism community. Autism.

[CR24] Leedham A, Thompson A, Smith R, Freeth M (2019). ‘I was exhausted trying to figure it out’: The experiences of females receiving an autism diagnosis in middle to late adulthood. Autism.

[CR25] Lewis LF (2017). A mixed methods study of barriers to formal diagnosis of autism spectrum disorder in adults. Journal of Autism and Developmental Disorders..

[CR26] Moss P, Howlin P, Savage S, Bolton P, Rutter M (2015). Self and informant reports of mental health difficulties among adults with autism findings from a long-term follow-up study. Autism.

[CR27] NICE. (2012). *Autism spectrum disorder in adults: Diagnosis and management.* London: National Institute of Clinical Excellence. Retrieved from https://www.nice.org.uk/guidance/cg142 32186834

[CR28] Orsmond GI, Shattuck PT, Cooper BP, Sterzing PR, Anderson KA (2013). Social participation among young adults with an autism spectrum disorder. Journal of autism and developmental disorders.

[CR29] Rogers CL, Goddard L, Hill EL, Henry LA, Crane L (2016). Experiences of diagnosing autism spectrum disorder: A survey of professionals in the United Kingdom. Autism..

[CR30] Russell G, Mandy W, Elliott D, White R, Pittwood T, Ford T (2019). Selection bias on intellectual ability in autism research: A cross-sectional review and meta-analysis. Molecular Autism..

[CR31] Shapiro, J. P. (1994). *No Pity*. Times Books.

[CR32] Shattuck PT, Narendorf SC, Cooper B, Sterzing PR, Wagner M, Taylor JL (2012). Postsecondary education and employment among youth with an autism spectrum disorder. Pediatrics.

[CR33] Sinclair J (1999) Why I Dislike ‘Person-first’ Language: Jim Sinclair’s website. Retrieved January 10, 2020, from https://web.archive.org/web/20090210190652/https://web.syr.edu/~jisincla/person_first.htm.

[CR34] Stagg SD, Belcher H (2019). Living with autism without knowing: receiving a diagnosis in later life. Health Psychology and Behavioral Medicine.

[CR35] Unigwe S, Buckley C, Crane L, Kenny L, Remington A, Pellicano E (2017). GPs’ confidence in caring for their patients on the autism spectrum: an online self-report study. British Journal of General Practice..

[CR36] Williams L, Hearst C (2019). Cat lady finds her way. Being Autistic: nine adults share their journeys from discovery to acceptance.

[CR37] Zuckerman KE, Sinche B, Mejia A, Cobian M, Becker T, Nicolaidis C (2014). Latino parents' perspectives on barriers to autism diagnosis. Academic Paediatrics.

